# Pregabalin treatment in a pregnant woman with glossopharyngeal neuralgia^☆^

**DOI:** 10.1016/j.bjorl.2017.01.010

**Published:** 2017-02-24

**Authors:** Azis Arruda Chagury, Karina Ribeiro Cavalcante Tavares, Raquel Marcon Camargo, Daniela Vieira Martins, Letícia Helena de Sousa Marques, Ali Mahmoud

**Affiliations:** a Universidade de São Paulo (USP), Hospital das Clínicas, Instituto Central, São Paulo, SP, Brazil; b Hospital Oftalmológico de Sorocaba, Banco de Olhos de Sorocaba (BOS), Sorocaba, SP, Brazil

## Introduction

Glossopharyngeal Neuralgia (GPN) is an unusual craniofacial clinical syndrome characterized by paroxysms of a stabbing pain in the distribution area of the glossopharyngeal nerve (IX) and sometimes the vagus (X) cranial nerve.^1^ It is characterized by severe paroxysmal pain typically on one side of the throat, ear, base of the tongue, and jaw angle. Pain attacks may be elicited by triggering stimuli, such as swallowing, coughing, talking or chewing. Glossopharyngeal neuralgia represents only 0.2%–1.3% of the facial pain syndromes^2^; however, it is often misdiagnosed as the more common Trigeminal Neuralgia (TN).

Treatment for glossopharyngeal neuralgia can be pharmacological or surgical. The first pharmacological line of treatment includes anticonvulsant medications such as carbamazepine, gabapentin, phenytoin or oxcarbazepine.^3^, ^4^ Surgical options should be considered in situations of drug intolerance, inefficacy, allergies or side effects associated with medical therapy.

Recently, pregabalin, another Gamma-Aminobutyric Acid (GABA) analog, has also been shown to be successful for the treatment of TN and GPN.^5^ Like gabapentin, it has a relatively benign side-effect profile, most commonly, dizziness, drowsiness, dry mouth, and weight gain.

## Case report

The study has been approved by the local ethics committee and written informed consent was obtained from the patient.

A 34 year-old Brazilian woman in the 16th week of her second pregnancy started experiencing strong intensity (Visual Analog Scale – VAS 9) intermittent odynophagia episodes lasting from seconds to hours, radiating to the right ear, and worsening with chewing and swallowing. It was assessed in the emergency room and a right ear wash was performed. Amoxicillin and paracetamol were prescribed for 7 days with no improvement.

The patient was referred to a specialist clinic with persistence of pain. On physical examination with otoscopy, an intact tympanic membrane was noted without bulging or redness. Grade II (Lynda-Brodsky classification) tonsils without purulent exudate or hyperemia were noted at oroscopy. There was no crackling of the temporomandibular joint and the patient had not undergone irradiation treatment to the hemiface. There were no comorbidities or drug allergies and the patient reported no previous history of this condition. Prednisone 20 mg/day was prescribed for 5 days but yielded no improvement.

Treatment was started with pregabalin 75 mg/day for 5 days and this was then increased to 150 mg/day. She returned after 15 days with gradual recovery from pain (VAS 7) and after 30 days suffered little discomfort (VAS 2). After 60 days of treatment, improvement was complete. She then followed a regular obstetric routine. Subsequently, her baby was born by cesarean delivery without complications or congenital changes.

In order to rule out a tumor as a possible etiology, encephalic and cervical magnetic resonance imaging (MRI) scans were taken after the gestational period and not before. This was because of the exposure risks that the contrast could cause to the fetus, and in any case, resolution of any tumor mass would only be performed in the postpartum period.

## Discussion

GPN is a rare disorder for which limited data are available in the literature. The treatment of choice is anticonvulsant medication.

Pregnancy is a state where there are pharmacokinetic changes affecting drug absorption, distribution, metabolism and elimination. These effects vary depending on the type of Antiepileptic Drug (AED), dose, period of pregnancy, but principally on the individual concerned. The AEDs can be teratogenic, and increase the risk of congenital malformations as well as adverse cognitive outcomes.^6^

The objectives of patient management during pregnancy are to maintain control of the disease and at the same time keep exposure to potentially teratogenic drugs to a minimum.^6^ Therefore, clinicians have to balance potential adverse effects to the fetus against the risks of uncontrolled maternal disease.^7^

The older-generation AEDs (e.g. phenobarbital, phenytoin, carbamazepine and valproic acid) are associated with increased rates of birth defects, retarded intrauterine growth and possibly also postnatal developmental delay. The new AEDs (felbamate, gabapentin, lamotrigine, levetiracetam, oxcarbazepine, pregabalin, tiagabine, topiramate, vigabatrin and zonisamide) have been introduced in the last 15 years, although clinical data on the teratogenic potential of these newer-generation AEDs are still scarce.^6^

Pregabalin is widely prescribed to treat bipolar disorder, neuropathic pain, generalized anxiety disorder and migraine.^8^, ^9^ It is extensively and rapidly absorbed, it does not bind to plasma proteins, and is excreted virtually unchanged by the kidneys which provides some confidence in its use during pregnancy compared to other AEDs.^6^

The second-generation AEDs are better tolerated, less prone to drug interactions and have more predictable pharmacokinetics than the first-generation AEDs, but they are not more effective.^10^ Older-generation antiepileptic drugs have been linked to some specific birth defects, such as spina bifida and orofacial clefts. Other changes reported in the literature with regard to the use of these medications include growth restriction and effects on cognitive development and behavior. There are reports that the newer antiepileptic drugs such as pregabalin have a low risk of major birth defects in monotherapy or polytherapy.^10^

Some animal studies with AEDs have reported skeletal malformations, neural tube defects, increased rates of spontaneous abortions, growth retardation, and behavioral anomalies.^9^ A recent publication on humans did not find a significantly increased malformation rate but observed an increased risk for low birth weight and preterm births compared to a general control group.^11^

In this study, choosing pregabalin for treatment was based on recent reports that have shown good results when treating neuropathic pain, especially trigeminal neuralgia, in addition to having minor side effects compared to other classes of anticonvulsants.^8^

Pregabalin (PGB) has a mechanism of action similar to Gabapentin (GBP), binding to calcium channels and modulating calcium influx as well as influencing GABAergic neurotransmission. This mode of action confers antiepileptic, analgesic and anxiolytic effects. It is more potent than GBP and can therefore be used at lower doses. Pregabalin, as Lyrica™, is licensed for treatment of peripheral and central neuropathic pain in adults.^12^ As renal blood flow increases by 50%–80% during pregnancy, serum concentrations of GBP or PGB may fall considerably. However, no systematic studies on the pharmacokinetics of GBP or PGB during the course of pregnancy have been published so far.^8^

According to the FDA, pregabalin is classified into category C for use in pregnancy. There are insufficient data on its use in pregnant women; the potential risk to the human fetus is unknown, therefore, pregabalin should not be used during pregnancy unless the benefit to the mother clearly justifies the potential risk to the fetus.

Detailed information was provided to the patient on the risks and benefits of her specific AED treatment as suggested in reported guidelines.^10^ After delivery, the mother and child were monitored during breastfeeding, as suggested in the literature, and the newborn developed as expected.^10^

As a rule, it is not considered ethically acceptable to conduct randomized controlled trials of drugs in pregnant women. Therefore, in most cases, the only available evidence on the safety of using drugs during pregnancy is from non-clinical studies of reproductive toxicity in animals. Thus, in most cases, the initial safety information during human pregnancy comes from empirical evidence. It should be remembered that there are factors other than drug dose, gestational age and duration of treatment which can modulate risk to the fetus, such as exposure to viruses, tobacco, or alcohol and which can pose an even greater risk to the fetus than any drug remedies applied.^13^ With regard to the case described in this study, therapeutic doses of pregabalin were administered and only for a short period.

## Conclusion

There are no reports in the literature on glossopharyngeal neuralgia in pregnant women. Based on the response of our patient, treatment with pregabalin seems to be a good option for pain symptom remission as an alternative effective therapy for the patient and with no apparent complications for the fetus.

## Conflicts of interest

The authors declare no conflicts of interest.
